# New perspectives on the progression of pulmonary fibrosis: the cascade from aberrant microvascular endothelial cell activation to fibrosis

**DOI:** 10.3389/fmed.2025.1639043

**Published:** 2025-08-21

**Authors:** Jie Zhou, Xiuwen Xia, Xing An, Danping Liu, Hongyi Zhao, Zengtao Sun, Weihong Li, Qingsong Huang

**Affiliations:** ^1^Clinical Medical College, Chengdu University of Traditional Chinese Medicine, Chengdu, Sichuan, China; ^2^Department of Respiratory Medicine, Hospital of Chengdu University of Traditional Chinese Medicine, Chengdu, Sichuan, China; ^3^School of Basic Medicine, Chengdu University of Traditional Chinese Medicine, Chengdu, Sichuan, China; ^4^Sichuan College of Traditional Chinese Medicine, Mianyang, Sichuan, China

**Keywords:** vascular endothelial cells, pulmonary capillary homeostasis, vascular remodeling, therapeutic strategies, pulmonary fibrosis

## Abstract

Traditional studies of pulmonary fibrosis (PF) have focused on alveolar epithelial cells injury and abnormal myofibroblast aggregation, but recent studies have revealed that imbalances in pulmonary capillary homeostasis also play pivotal roles in this disease. The pulmonary microvasculature, composed of aerocyte capillary (aCap) and general capillary (gCap) endothelial cells, forms the core structure of the alveolar-capillary membrane. It performs key roles in gas exchange and nutrient/metabolite transport, while modulating the trafficking of inflammatory factors and immune cells and regulating alveolar damage repair. Abnormal activation of pulmonary microvascular endothelial cells in pulmonary fibrosis, reprogramming of cellular metabolism, secretion of proinflammatory and profibrotic factors, and disruption of pulmonary capillary homeostasis, lead to abnormal remodeling of the pulmonary microvasculature and other pathological changes, promoting the deterioration of PF. Notably, maintaining and restoring normal pulmonary capillary homeostasis is beneficial for improving the local microenvironment of fibrotic lesions and attenuating pathological changes such as hypoxia. In this review, the pathological changes associated with pulmonary capillary homeostasis imbalance in PF are described. Therapeutic directions for restoring pulmonary capillary homeostasis are also proposed with the expectation that they will provide assistance in the treatment of PF.

## 1 Introduction

Interstitial lung diseases (ILDs) are characterized by inflammation or fibrosis of the lung parenchyma. ILD with fibrosis as the predominant pathological manifestation may be classified as secondary or idiopathic. Common causes of secondary ILD include connective tissue disease-associated ILD (e.g., rheumatoid arthritis, scleroderma), environmental/occupational exposure-related ILD (e.g., silicosis, asbestosis), and drug-induced ILD (e.g., amiodarone, bleomycin), among others (1). Idiopathic pulmonary fibrosis (IPF) is the most important subtype of ILD, and accounts for approximately one-third of ILD patients (2). The incidence of IPF varies according to region, with 7–1,650 IPF cases per 100,000 people worldwide, and the annual incidence of IPF is increasing (3–5). IPF has a high mortality rate, a life expectancy of 2–3 years (6), and a lack of effective treatments. Pirfenidone and nintedanib are approved antifibrotic drugs that can slow the decline in lung function in IPF but do not reverse pulmonary fibrosis (7, 8). And Long-term use of these drugs has a high incidence of adverse events, such as gastrointestinal events (dyspepsia, diarrhea, etc.), skin-related events (rash, photosensitivity reactions, etc.), and in severe cases, discontinuation is required due to intolerable adverse events (9–13). The cost of treating IPF is much greater than that of the general population because of the long treatment period, which imposes a significant financial burden on the families of IPF sufferers and poses a significant challenge to global public health (14–16). This is due to the complexity of the pathogenesis of IPF, which hinders the development of effective therapeutic options.

Previous studies have suggested that dysregulation of alveolar epithelial cells (AECs) injury and repair, and overproduction of myofibroblasts are the central mechanisms underlying the emergence of pulmonary fibrosis (PF) (17). However, this does not explain the pathological changes in PF lesions, where the density of pulmonary capillaries decreases or disappears. Furthermore, 16% of myofibroblasts in PF lesions are derived from vascular endothelial cells (VECs) (18). This evidence suggests that the role of VECs in PF has been overlooked (19, 20). An analysis of VECs in fibrosis revealed that abnormal activation of VECs stimulated by pathological factors leads to structural and functional alterations in the cells, disrupting pulmonary capillary homeostasis and leading to pathological alterations in the vasculature, such as increased permeability and vascular remodeling (21, 22). Moreover, an imbalance in pulmonary microvascular homeostasis disrupts alveolar–capillary gas exchange function (19). Therefore, this review summarizes the specific pathological mechanisms by which the abnormal activation of pulmonary microvascular endothelial cells (PMVECs) disrupts pulmonary capillary homeostasis and promotes the progression of PF. And it proposes a therapeutic strategy to restore pulmonary capillary homeostasis for the treatment of PF, which provides ideas for the development of new therapeutic options.

## 2 The normal structure and function of PMVECs are fundamental to the maintenance of pulmonary capillary homeostasis

**Figure 1:**
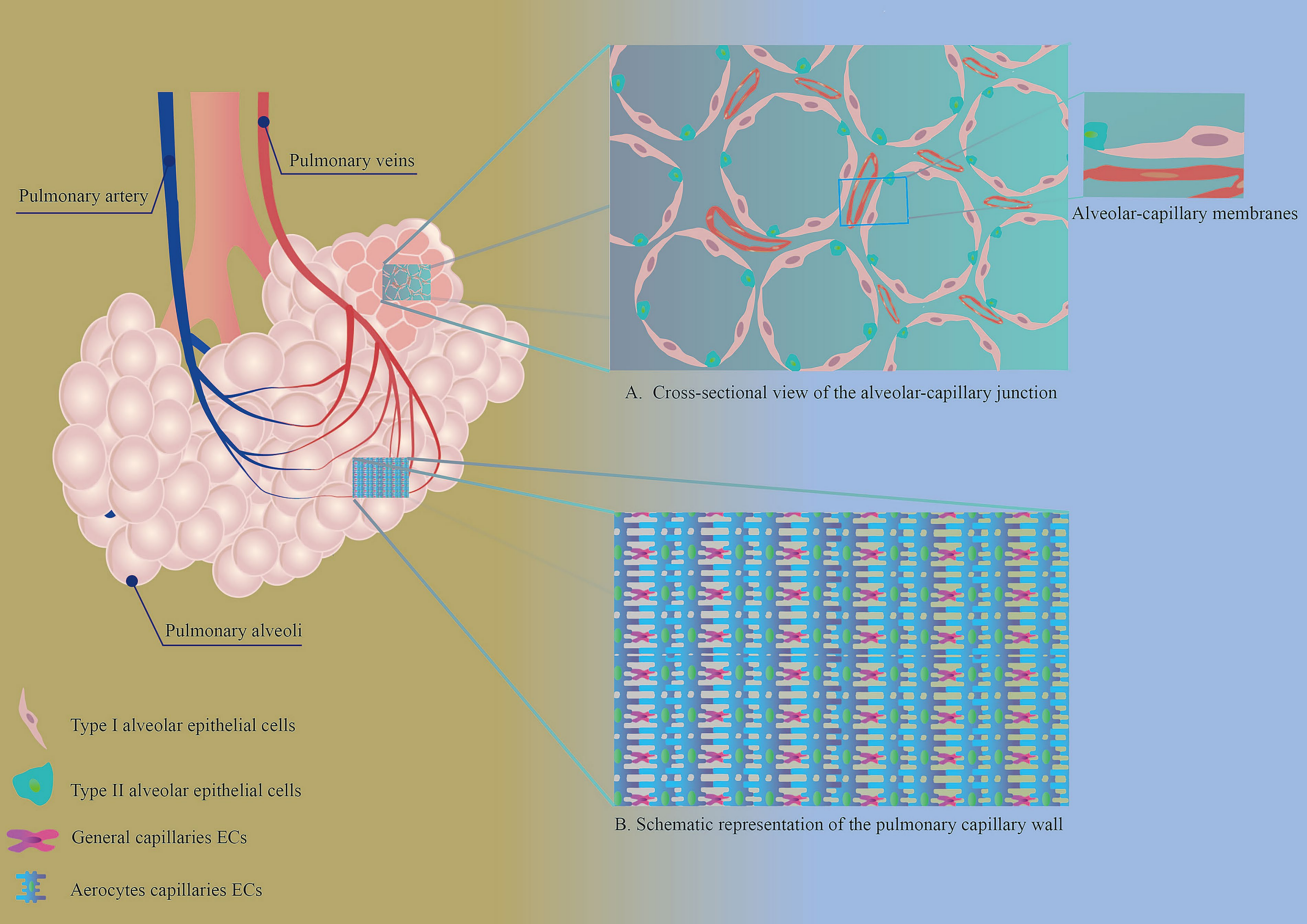
**(A)** Cross-sectional view of the alveolar-capillary junction. AECs and PMVECs make up the alveolar-capillary membrane, an important structure for gas exchange in the lungs. **(B)** Schematic representation of the pulmonary capillary wall. The walls of healthy pulmonary capillaries are formed by two distinct endothelial cell types: aerocytes and general capillary endothelial cells.

Pulmonary capillaries are vascular barriers formed by the interconnection of VECs, which control the entry and exit of nutrients, metabolic products, cells, etc. When lung tissue is damaged, the vascular barrier also allows cytokines and immune cells, among others, to enter the damaged area and participate in the inflammatory response, among others (23). Pulmonary capillaries are closely connected to alveoli, forming an alveolar–capillary membrane structure (Figure 1A), which facilitates gas exchange between the lungs and the external environment. Pulmonary capillaries are composed of two types of VECs (Figure 1B) (24–26). The first type is aerocyte capillary (aCaps) ECs, which are responsible for gas exchange and cellular transport within the lungs. The second type consists of general capillary (gCaps) ECs, which have a progenitor cell function and are involved in processes such as vascular repair, immunomodulation and maintenance of capillary homeostasis. Single-cell analysis revealed that in the healthy state, aCap and gCap ECs were stable, and only a very small number of gCap ECs intermittently differentiated into aCap ECs (26). This study also found that gCap ECs could differentiate into aCap ECs in the injured state, but the exact differentiation process was not explained. Subsequent single-cell transcriptome profiling revealed that after damage to the pulmonary capillary endothelium, gCap ECs appeared as a new population expressing apelin and the stem cell marker protein C receptor, and then continued to transform into proliferative endothelial progenitor-like cells expressing the apelin receptor and the pro-proliferative transcription factor Foxm1, which rapidly replenished depleted ECs, including the highly specialized aCap ECs (27).

## 3 Abnormal activation of PMVECs disrupts pulmonary capillary homeostasis and promotes the progression of PF

**Figure 2:**
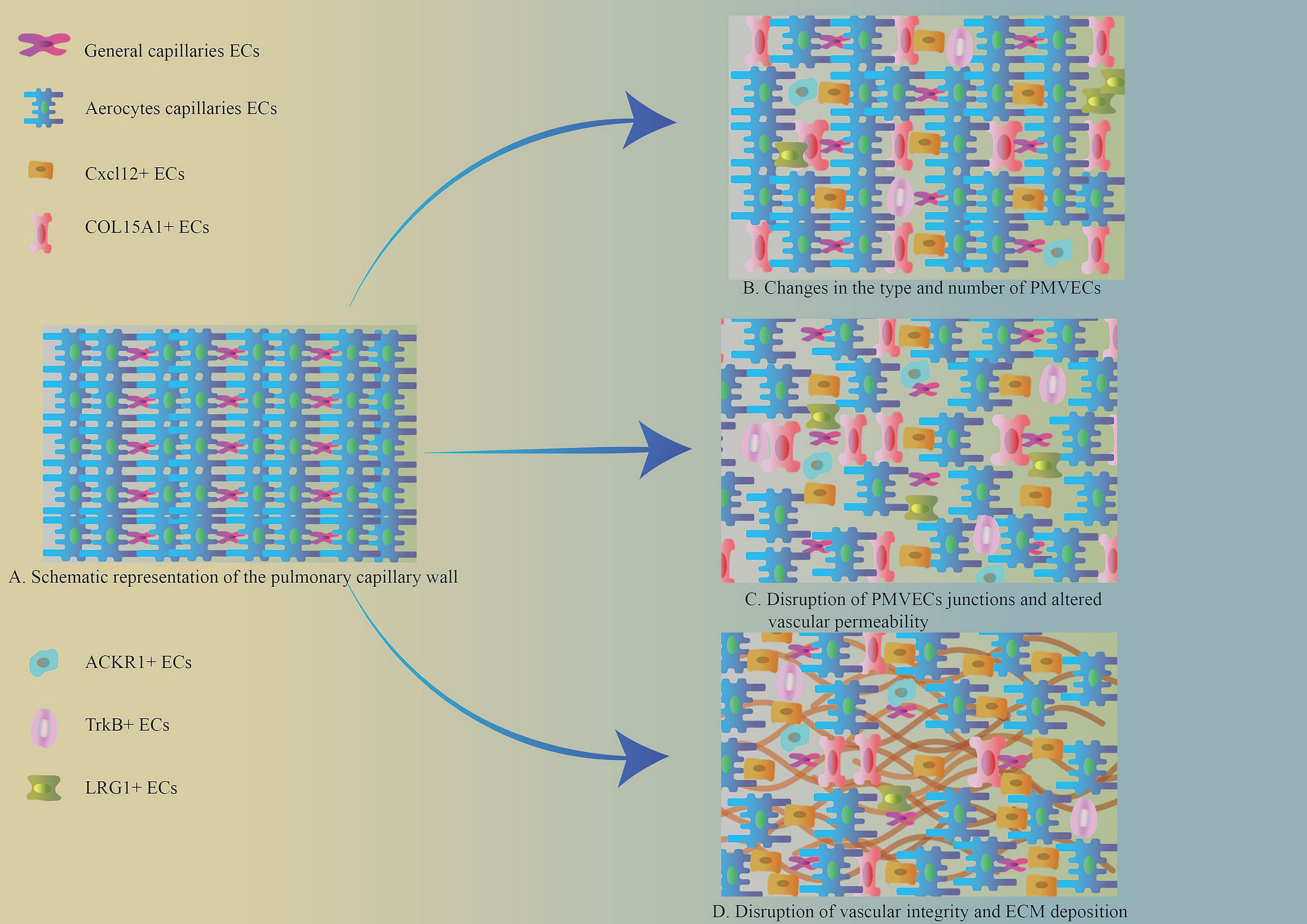
**(A)** Schematic representation of the pulmonary capillary wall. **(B)** Changes in the type and number of PMVECs. The number of pulmonary microvascular gCap ECs was significantly reduced in the area of PF. And new VEC phenotypes appeared, including Cxcl12^+^, ACKR1^+^, TrkB^+^, LRG1^+^, and COL15A1^+^ phenotypes. **(C)** Intercellular junctions of PMVECs in the region of pulmonary fibrosis lesions were disrupted, vascular permeability was increased, and the barrier function of blood vessels suffered disruption. **(D)** Disruption of vascular integrity and ECM deposition. Pulmonary capillary permeability is altered in the area of pulmonary fibrosis lesions, and some VECs produce large amounts of ECM via EndMT, which promotes lung fibrosis progression.

Normal VECs are usually in a homeostatic state and are transiently activated in response to stimulation by injurious factors, and return to the homeostatic state after the injury has been repaired (Figure 2A). Single-cell RNA sequencing further demonstrates that the activation of VECs is reversible; for example, in young mice, after bleomycin stimulation, activated VECs return to a resting state after completion of repair (28). However, in pathological conditions, such as persistent fibrosis, this leads to sustained aberrant activation of VECs. PMVECs showed persistent activation in response to stimulation by pathogenic factors (Table 1). Moreover, single-cell RNA sequencing showed that PMVECs are activated to undergo pro-fibrotic changes at an early stage of PF (21, 29).

**Table 1:** Triggers of PMVECs activation.

Sources of triggers	Precipitating factor	Pathway/mode of activation	References
*In vitro* factors	Radiation	Activation of ubiquitin-specific peptidase 11	(110–112)
	Dust (silica, Silicosis, PM2.5, etc.)	Decreased expression of LncRNA Gm16410	(95, 113–117)
		Down-regulation of NOX2 protein expression and overexpression of CAT protein promote intracellular reactive oxygen species accumulation	
		Overexpression of ZC3H4 promotes endoplasmic reticulum stress and autophagy	
		Increasing circHECTD1 expression and thus inhibiting HECTD1 protein expression	
		Overexpression of the transcriptional regulator CEBP3	
	Volatile organic compounds	Suppression of Atf3 gene and promotion of Gas6 overexpression	(96)
	Viruses (COVID-19, Influenza A virus, etc.)	PD-L1, IDO and STAT3 were abnormally expressed	(64, 105, 118–121)
		Promotion of GRK2 overexpression that inhibits S1PR1 protein expression	
		Activation of intercellular adhesion molecule-1	
		Overexpression of phosphodiesterase type 5	
*In vivo* factors	Heredity	Rare Variants in Telomere Maintenance and Surfactant Protein Genes	(122)
	Aging	Cellular senescence or premature senescence	(58, 95)
		Loss of ERG function	
	Disease	Reactive oxygen species generation and transglutaminase (TGase) activation	(123)
Pathological changes of adjacent cells	AECs	Caveolin-1 was overexpressed	(124)
	Fibroblasts	Secretion of cytokines	(98)

### 3.1 Aberrant activation of PMVECs in PF lesions alters their cytoarchitecture and disrupts vascular homeostasis

The cytoarchitectural alterations of PMVECs in PF are mainly reflected in the altered number and abnormal distribution of VECs subpopulations, disruption of the connective structures between VECs, and endothelial mesenchymal transition (EndMT). These pathological changes lead to an imbalance in pulmonary capillary homeostasis, increasing vascular permeability and driving abnormal vascular remodeling in PF.

#### 3.1.1 Altered subpopulation numbers and abnormal distribution of PMVECs

PMVEC subpopulations and numbers were different in healthy and fibrotic lung tissues (Figure 2B). Typical gCap capillary endothelial cell numbers were significantly reduced in lung fibrotic tissues (19, 30, 31). Phenotypic changes in activated pulmonary capillary endothelial cells occur under the influence of the fibrotic environment of the lung. Single-cell RNA sequencing of different phenotypes of PMVECs differentiated them, and typical phenotypes included Cxcl12^+^, ACKR1^+^, TrkB^+^, LRG1^+^, and COL15A1^+^. The Cxcl12^+^ subpopulation was associated with various pro-fibrotic activities, including inflammation, vascular remodeling, and ECM deposition (21). The ACKR1^+^ subpopulation is distributed within the veins and is involved in the regulation of inflammatory pathways, pulmonary vein remodeling and angiogenesis-related pathways, and is closely associated with αSMA^+^ mesenchymal cells (28, 32, 33). The presence of TrkB^+^ subpopulation marks the activation of capillary ECs, is predominantly located in areas where fibroblasts accumulate after lung tissue injury, and correlates with the severity of PF (28). LRG1^+^ subpopulation interacts with lung fibroblasts through the TGFβ/Smad2 pathway, and promotes ECM deposition (34). COL15A1^+^ VECs are located in the blood vessels surrounding the proximal fine bronchioles in healthy lung tissue. However, in IPF, a large number of COL15A^+^ VECs were abnormally distributed in fine bronchioles and fibrotic areas (35, 36).

#### 3.1.2 Disruption of VECs junctions and increased vascular permeability in PF lesions

Normal VECs make up the vascular barrier by means of tight junctions, adherent junctions, and gap junctions (Figure 1B) (37). This gives the vasculature the ability to selectively pass metabolic substances and cells. In PF lesions, the connective structure between PMVECs is disrupted (Figure 2C) (38), the barrier function of the vasculature is impaired, and vascular permeability within the lesion is increased, leading to local inflammation. Sphingosine-1 phosphate (S1P) in phospholipid membranes plays an important role in maintaining the connections between PMVECs. Under normal conditions, S1P maintains the connectivity between lung capillaries (39). When vascular endothelial junctions are disrupted, the overexpression of S1P restores endothelial cell junctions and strengthens the endothelial barrier function (40–42). Decreased expression of S1P was observed in PF, along with increased levels of ceramide, which has a disruptive effect on intercellular junctions and disrupts the integrity of the vascular endothelium (43).

#### 3.1.3 EndMT disrupts vascular integrity and promotes perivascular extracellular matrix protein deposition

PMVECs can be activated into mesenchymal cells with ECM secretion after lung tissue injury, a process known as EndMT (36, 44), which is one of the key pathological changes that promote the exacerbation of PF (Figure 2D). Persistent endothelial cell activation is prevalent in pulmonary fibrosis lesions (28, 45). Recently, it has been found that there is a transient acquisition of mesenchymal characteristics after Plvap^+^ gCap endothelial cell activation in PF, while still maintaining endothelial properties (46). As fibrosis worsened, endothelial cell activation became more frequent. This better explains the course of pathological changes of PMVECs in PF. With the accumulation of inflammation (IL-1β, TNF-*α*, etc.), pro-fibrotic factor (TGF-β1) and other cytokines in fibrotic lungs, the microenvironment around PMVECs is altered (47–49). This leads to an increased susceptibility of PMVECs to fibrosis, and transient EndMT promotes vascular repair. However, as fibrosis progresses, processes such as iron death, glycolysis, and lipid metabolism are altered in PMVECs (50, 51), promoting increased expression of sterol regulatory element-binding protein 2 (SREBP2) (a key protein for cholesterol homeostasis), the transcription factors Sox9 and Snail, and ultimately leading to persistent endothelial cell activation (47, 52, 53). And it induces EndMT in the ECs of neighboring lung microvessels, leading to over-repair of lung capillaries, disruption of their integrity, increased vascular permeability, and the appearance of a distinct honeycomb structure (54–56).

### 3.2 Abnormal activation of VECs in PF alters their cellular function and promotes the formation of a local inflammatory environment and fibrotic lesions

PMVECs in the physiological state are associated with the intrinsic immune response, intercellular adhesion and endothelial regeneration (21, 57). In contrast, in PF, activated PMVECs are involved in the inflammatory response and fibrosis, and are also involved in coagulation processes. Some activated PMVECs exhibit reduced endothelial-specific gene expression and increased expression of inflammation-related genes (58, 59), secrete large amounts of inflammatory factors (Table 2) and form a local inflammatory microenvironment.

**Table 2:** Inflammatory and profibrotic factors secreted by PMVECs.

Categories	Cytokines	Function	References
Inflammatory factors	CXCL12	CXCL12-CXCR4 axis is involved in inflammation, immunity, EndMT, angiogenesis.	(125, 126)
	CXCL10	Involved in inflammation response.	(58)
	IL-6	Alteration of vascular permeability via JAK/STAT3 pathway, MEK/ERK pathway	(127)
	TNF-α	It is involved in innate immune response and inflammatory response.	(58)
	INF-γ		
Profibrotic factors	TGF-β	It promotes fibrotic processes such as EndMT.	(61, 112, 128, 129)
	CTGF	Synergistic TGF-*β*1 promotes fibrosis progression.	(50, 61, 128, 130, 131)
	PDGF	PDGF-C acting on ECs promotes fibrosis.	(61)
	IL-1α	IL-1α secreted byECs promotes ECM production.	(132)
	Endothelin-1 (ET-1)	Promotes TGF-β1 production and synergises its profibrotic effects.	(129, 133)
	IL-11	Promotion of EndMT.	(134)
	MMP-19	Synergises with ET-1 to promote EndMT; recruits monocytes.	(135)

Peripheral immune cells, including macrophages and monocytes, are also recruited to amplify the inflammatory response (60). In addition to the increased expression of inflammatory genes, this fraction of cells also overexpresses profibrotic genes, promoting the deterioration of pulmonary fibrotic lesions (61), as shown in Table 2. Microvascular thrombus formation has also been observed in damaged pulmonary capillaries and is associated with VEC injury, leading to the release of anticoagulant molecules and increased levels of procoagulant factors on the vascular surface (50, 62). Microthrombi also slow local blood flow, exacerbate local thrombus formation, lead to a localized hypoxic state in the lesion, promote the expression of inflammatory and fibrotic genes in the pulmonary capillary endothelium, and recruit immune cells, among other types of cells (63).

## 4 Imbalances in pulmonary capillary homeostasis promote pulmonary capillary remodeling and ECM protein deposition and attenuate lung tissue repair

**Figure 3:**
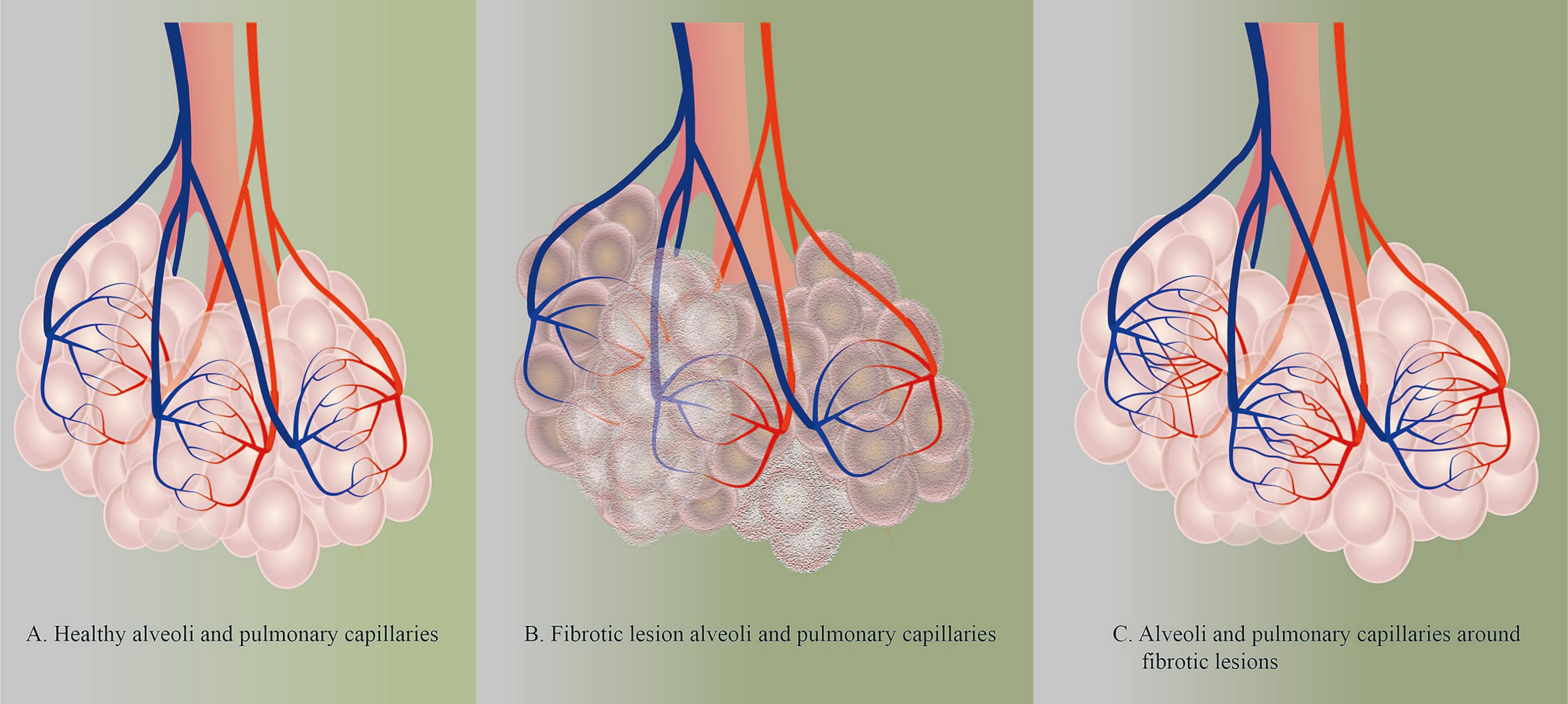
**(A)** Healthy alveoli and pulmonary capillaries. **(B)** Fibrotic lesion alveoli and pulmonary capillaries. ECM protein deposition and reduced density of PMVECs within fibrotic lesions in lung tissue with PF. **(C)** Alveoli and pulmonary capillaries around fibrotic lesions.

Metabolic reprogramming occurs in pulmonary microvascular endothelial cells in pulmonary fibrotic lesions, which disrupts the balance between damage to and repair of the pulmonary capillaries and changes vascular permeability within the lesion, leading to pathological changes such as hypoxia, inflammatory infiltrates, and ECM protein deposition in the lesion. Pulmonary capillaries, in turn, undergo vascular remodeling (Figure 3) (20, 30). In the early stages of pulmonary fibrosis, pulmonary capillaries exhibit reduced integrity and increased permeability (64, 65). With the abnormal repair of pulmonary fibrosis lesions, the distribution of blood vessels within the lesion area decreases, whereas the density of blood vessels increases in the area surrounding the lesion (66–68). In the end stage of pulmonary fibrosis, because of the expansion of fibrotic lesions, the cross-sectional area of pulmonary capillaries within the lesions decreases or even disappears, leading to an increase in pulmonary circulatory resistance and even pulmonary hypertension (69, 70).

### 4.1 Vascular homeostatic imbalance in PF results in the disappearance of pulmonary capillaries within the lesion and an increase in the density of pulmonary capillaries around the lesion

Vascular injury and regenerative imbalance in PF are central to pulmonary capillary remodeling. Pulmonary capillaries show different pathological manifestations at different stages of PF. As PF progresses, there is a gradual decrease in capillary density within the lesion and a lack of vascular structures within the mature fibrotic lesion (Figure 3B) (30). This phenomenon is associated with increased expression of vascular inhibitory factors (e.g., PEDG) and decreased expression of angiogenic factors (e.g., VEGF) and vasculoprotective factors (e.g., BMPR2) in lesions (67). PEDG inhibits the expression of VEGF in lesions and induces apoptosis in VECs, which results in undetectable low levels of VEGF in lesions (66, 67, 71). Moreover, in the microenvironment of fibrosis, the expression of BMPR2, which is protective for endothelial cells, is reduced, increasing the susceptibility of the vascular endothelium to fibrosis (72).

In PF, in contrast to the situation within fibrotic lesions, VEGF proteins were detected in the vascular endothelium within nonfibrotic lesions (67, 71). These VEGFs are mainly due to the activation of the HIF-*α* pathway by hypoxic vascular endothelial cells, which initiates VEGF transcription and expression (73, 74). This process is a compensatory manifestation of the pathology. In addition, the reduced vascular density within the lesion leads to an increase in fluid shear stress in the blood around the lesion, which stimulates endothelial cells to produce miR-143-3p and promotes capillary regeneration in healthy lung tissue (75). In addition to the role of VECs in angiogenesis, the upregulation of proangiogenic genes was also observed in the gene expression profile of airway epithelial cells (76). Furthermore, recent studies have shown that a subpopulation of myofibroblasts characterized by the expression of collagen triple helix repeat containing 1 (CTHRC1) exists in PF (77–81). These cells are derived from alveolar fibroblasts and can express high levels of ECM (82–86). In tumor-related studies, CTHRC1 protein promotes vascular remodeling and angiogenesis by enhancing glycolytic processes in VECs (87, 88). This suggests a potential mechanism whereby CTHRC1^+^ fibroblasts may contribute to the increased capillary density around fibrotic lesions, representing a promising future research direction. Together, these factors contribute to the emergence of newborn pulmonary capillaries around the lesion and the increased percentage of VECs in the PF (Figure 3C) (89). Thus, protection of pulmonary capillaries in the lesion helps delay the onset of pulmonary vascular remodeling and increases the time needed for the repair of damaged lung tissue.

### 4.2 Imbalances in vascular homeostasis within pulmonary fibrosis lesions reduce alveolar repair capacity and increase ECM protein deposition

The essence of PF is the deposition of ECM proteins due to excessive repair. More studies have suggested that PF begins with dysregulated damage and repair of AECs. Under normal conditions, PMVECs can secrete S1P or perform paracrine delivery of miR-200c-3p, which promotes the differentiation of AT2 cells into AT1 cells to repair damaged alveoli (60, 90). It can also secrete MMP-14 to promote the repair of AECs (91). However, in pulmonary fibrosis lesions, MMP-14 and miR-200c-3p expression was reduced in damaged PMVECs, which attenuated the repair capacity of damaged alveoli (92). In addition, pulmonary capillaries suffer damage in the early stage of fibrosis, resulting in increased vascular permeability, plasma exudation into the interalveolar stroma and alveolar lumen, and ultimately, the formation of hyaline membranes covering the surface of the alveolar epithelium, which affects the gas exchange capacity of alveolar capillaries (93). Thus imbalances in pulmonary capillary homeostasis can attenuate the repair capacity of damaged alveoli.

In PF, damaged PMVECs can activate the proliferation and differentiation of fibroblasts through multiple pathways. Changes in the content of proteins secreted by damaged PMVECs influence lung fibroblasts to develop a fibrotic response, such as decreased expression of ERG and BMPR2 or increased expression of CTGF in endothelial cells, which can lead to fibroblasts expressing a fibrotic phenotype (58, 72, 94). Some PMVECs with reduced expression of the chemokine receptor CXCR7 were recruited toward perivascular macrophages. This resulted in sustained upregulation of Jagged1 (ligand for Notch) on PMVECs, activating the Notch signaling pathway in perivascular fibroblasts (60). At the same time, Galectin-3 (Gal3) secreted by senescent PMVECs can initiate fibroblast-myofibroblast differentiation by binding to TGFBR1 on the cell membrane of lung fibroblasts (95). In addition, Gas6, secreted by PMVECs with a PANoptosis phenotype, binds to Axl in fibroblasts and activates fibroblasts (96). These molecular pathways demonstrate how aberrant PMVECs signaling directly promotes pathogenic fibroblast transitions and ECM deposition.

## 5 Therapeutic strategies to restore pulmonary capillary homeostasis in PF

The maintenance of pulmonary capillary homeostasis is the basis for the exchange of gasses, nutrients and metabolites between the blood and alveoli. In PF lesions, the structure and function of VECs are highly abnormal. Maintaining and restoring normal pulmonary capillary homeostasis is conducive to attenuating pathological changes such as hypoxia in fibrotic lesions, as well as increasing the efficiency of drug delivery and ameliorating PF (65, 97). Therefore, to restore pulmonary capillary homeostasis, damaged PMVECs can be repaired by improving the inflammatory and fibrotic microenvironments around PMVECs and increasing the resistance of endothelial cells to fibrotic alterations.

The first step is to improve the microenvironment. Structural and functional changes in PMVECs during fibrosis are strongly linked to the surrounding inflammatory and fibrotic environment. Because it is not possible to isolate the communication between endothelial cells and the surrounding environment, the microenvironment can be improved by inhibiting the secretion of factors with damaging effects or by increasing beneficial factors in the microenvironment. Myofibroblasts, the core cells involved in the development of pulmonary fibrosis, can secrete large amounts of profibrotic cytokines. A team developed an engineered mesenchymal stem cell (MSC) called MSC-MM@LPHN to target myofibroblasts in lung tissues by modifying the surface of MSCs to encapsulate ROS-responsive paper polymer hybrid nanoparticles of metformin and macitentan, which induced their dedifferentiation, reduced endothelial damage factor secretion and restored vascular homeostasis (98). Thrombopoietin mimetic (TPOm), which acts on the TPOm receptor, inhibits ICAM-1 expression in primary mouse PMVECs, reducing endothelial cell–neutrophil adhesion and decreasing immune cell recruitment (99). Another study inhibited iron death and fibrotic alterations in endothelial cells by increasing dopamine in the periendothelial environment and balancing lipid/glucose metabolism in endothelial cells (51).

The next step is to repair damaged PMVECs. Maintaining the normal differentiation of gCaps repaired damaged lung capillaries and restored vascular homeostasis. Matrix Gla protein (MGP), an antagonist of bone morphogenic protein (BMP), is highly expressed in lung cells (100, 101), and MGP supports the normal differentiation of progenitor cells and inhibits the abnormal differentiation of endothelial cells (102, 103). However, the mechanism by which MGP promotes the differentiation of gCaps ECs to repair damaged pulmonary capillaries in PF needs to be further investigated. Moreover, MGP binds to BMP-1 and reduces the production of mature TGFβ1, thereby inhibiting EndMT (100). Treamid may be a promising antifibrotic drug that can stimulate regeneration of the lung endothelium in patients with IPF (104).

Finally, the resistance of PMVECs to fibrotic alterations is enhanced. In the lung fibrosis environment, PMVECs are susceptible to fibrotic stimuli. This is related to the fact that the stimulation of PMVECs in the fibrotic microenvironment leads to intracellular metabolic reprogramming, with alterations such as increased glycolysis and reduced expression of nicotinamide adenine dinucleotide and the stromal cell proteins CCN3 and S1PR1 (45, 105–108). Therefore, maintaining normal intracellular metabolic processes in PMVECs enhances their resistance to fibrotic alterations. For example, inhibition of CD38 gene expression can significantly affect fibrotic lesions during EndMT (45). The overexpression of S1PR1 can also increase the stability of connections between PMVECs and improve vascular permeability (105, 107). In PMVECs that have undergone fibrotic changes, the EndMT process can be inhibited by miR-218 in exosomes secreted from MSCs, which inhibits the MeCP2/BMP2 pathway (109). Therefore, enhancing the resistance of PMVECs to fibrotic alterations could inhibit pathological changes in the vasculature within pulmonary fibrotic lesions and protect the integrity of the vascular endothelium.

## 6 Conclusion

Abnormal activation of PMVECs disrupts pulmonary capillary homeostasis one of the core pathological mechanisms underlying the progression of PF. Abnormal activation of PMVECs disrupts the structure and function of normal cells, leading to disruption of intercellular junctions, altered vascular permeability, and imbalance of pulmonary capillary homeostasis. These pathological changes cause impaired substance exchange function, inflammatory response, abnormal ECM deposition and other pathological changes within the fibrotic lesions. This ultimately leads to abnormal vascular remodeling. Therefore maintaining or restoring pulmonary capillary homeostasis is conducive to ameliorating the above pathological changes, and improving the efficiency of drug delivery to fibrotic lesions, thereby inhibiting or reversing the progression of PF.
